# The effects of different types of Tai Chi exercise on anxiety and depression in older adults: a systematic review and network meta-analysis

**DOI:** 10.3389/fpubh.2023.1295342

**Published:** 2024-01-08

**Authors:** Xiaoqin Kuang, Yangjian Dong, Long Song, Lili Dong, Guodong Chao, Xinxin Zhang, Jiefu Yang

**Affiliations:** ^1^College of Physical Education and Health, Guilin University, Guilin, China; ^2^College of Physical Education and Health, Guangxi Normal University, Guilin, China; ^3^College of Physical Education, China Three Gorges University, Yichang, China

**Keywords:** Tai Chi, anxiety, depression, older adults, network meta-analysis

## Abstract

**Background:**

The body of evidence supporting the beneficial effects of Tai Chi in reducing anxiety and depressive symptoms in older adults is steadily increasing. Nonetheless, there remains a scarcity of studies directly comparing the clinical effectiveness of various Tai Chi exercises in addressing anxiety and depressive symptoms in older adults. Thus, this study aimed to systematically review and analyze the therapeutic efficacy of four types of Tai Chi interventions in enhancing anxiety and depressive symptoms in older adults.

**Methods:**

During the period up to July 26, 2023, searches were conducted in the Web of Science, PubMed, the Cochrane Library, CNKI, and the Chinese Scientific Journal Database (VIP). The language scope encompassed both English and Chinese. Two independent reviewers conducted the literature review and data extraction. Review Manager 5.4 was employed for traditional meta-analysis and risk assessment, while version 15 of STATA software was used for generating evidence and funnel plots for network meta-analysis. This study was registered in PROSPERO number CRD 42023442270.

**Result:**

In this analysis, a total of 20 studies were included, involving 1798 participants. The findings of the pairwise meta-analysis revealed that Tai Chi intervention was more effective than the control group in reducing anxiety (SMD: −1.19, 95% CI: −2.04, −0.34, *p* < 0.05) and depression (SMD: −0.65, 95% CI: −0.95, −0.65, *p* < 0.05) symptoms among older adults. The network meta-analysis specifically focused on anxiety symptoms and found that Yang-style Tai Chi (69.9%) had the most favorable outcome, followed by the 24-form Simplified Tai Chi (66.8%). In terms of depression symptoms, the Tai Chi Exercise Program (92.6%) had the highest performance, followed by Yang-style Tai Chi (77.9%).

**Conclusion:**

The findings of this study imply that Tai Chi can have beneficial outcomes in the reduction of anxiety and depressive symptoms among older individuals. Specifically, when examining various forms of Tai Chi interventions, it was observed that Yang-style Tai Chi exhibited a greater efficacy in alleviating anxiety symptoms, whereas Tai Chi exercise programs demonstrated a higher effectiveness in improving depressive symptoms. Nonetheless, it is strongly advised that older adults select an exercise program that aligns with their interests and preferences, as this can enhance social integration and overall well-being.

**Systematic Review Registration:**

https://www.crd.york.ac.uk/prospero/display_record.php?ID=CRD42023442270, identifier [CRD42023442270].

## 1 Introduction

With the improvement of global living standards, the world’s average life expectancy, the number of elderly people, and the proportion of the elderly population are all showing an increasing trend. More and more countries are experiencing the process of population aging (1). In the face of the accelerated pace of life and the increasing social pressures, older adults encounter challenges in dealing with physical limitations, disabilities, or other bodily issues caused by the decline in bodily functions. The lack of physical activity, such as sedentary behavior, may lead to complications such as cognitive decline, aging processes, and inflammation (2–5). Additionally, this situation is more likely to trigger psychological health problems. (6). According to data from the World Health Organization in 2017, the prevalence of anxiety disorder and depression among people aged 60 and above is 3.8 and 7%, respectively, (7), which has become one of the main causes of disability worldwide (8).

Anxiety and depression, as common psychological problems, have been increasing in prevalence. However, the current global treatment situation has not reached an ideal level (9, 10). Due to the complex mechanisms of these conditions, many patients can only rely on medication and cognitive behavioral therapy (11, 12). However, medication treatment has limitations and negative side effects, while cognitive behavioral therapy requires face-to-face treatment, which is not convenient for many patients. This is particularly challenging for the elderly, a vulnerable group in society, whose treatment prospects are even less optimistic. In addition, medication treatment and cognitive behavioral therapy also increase the cost of mental health care, imposing a significant economic burden on patients and their families. Therefore, the importance of choosing low-cost and side-effect-free physical exercise becomes particularly prominent (13). Many mild or moderate physical activities have become of popular choices for exercise due to their positive impact on mental health (14, 15), Tai Chi, as a moderate-intensity exercise, is favored by the elderly population for its gentle nature, lack of space limitations, and ease of learning (16). In clinical applications, Tai Chi has already demonstrated good effects in reducing anxiety and depression symptoms. For example, Research findings have indicated that the practice of Tai Chi has been associated with notable enhancements in both anxiety and depression symptoms among the elderly population (17, 18), and meta-analysis results also demonstrate its significant efficacy in improving anxiety and depression in the elderly (19, 20).

Tai Chi, being a traditional martial art, can be classified in various ways. Firstly, it can be categorized based on its founding families, which include Chen, Yang, Wu, Wu, and Sun. Secondly, it can also be classified according to its difficulty level, such as 8, 16, 24, 32, and 42. Additionally, Tai Chi comprises three postures: high, middle, and low. Each type of Tai Chi possesses distinct characteristics and requires different levels of physical strength and flexibility from its participants. Therefore, it is crucial to choose a Tai Chi style that suits the condition of elderly individuals with chronic illnesses or disabilities and to practice under the guidance of professionals. For instance, lower postures demand higher levels of strength, flexibility, and balance. As a result, beginners are advised to commence their Tai Chi practice with higher postures and gradually progress to lower ones (21).

However, it is precisely because of the diversity of Tai Chi forms, including exercise frequency, duration, and target population, that there is inconsistency. At the same time, different Tai Chi exercise methods will also produce different effects. Therefore, there is currently no definite answer in choosing the exercise method suitable for the elderly population, and there is still uncertainty about which therapy is more suitable for elderly patients. Additionally, a majority of RCTs and meta-analyses are limited to comparing the therapeutic effects of Tai Chi with control groups, without comparing the intervention effects of different forms of Tai Chi. Compared to traditional meta-analysis, network meta-analysis (NMA) can incorporate multiple intervention measures and rank them to determine the optimal intervention strategy (22, 23). Therefore, this study employed network meta-analysis and utilized indirect comparison methods to quantitatively compare the effectiveness of different intervention measures in treating similar cases. Additionally, a hierarchical ranking of various intervention measures was conducted to select the best treatment plan (24). This study aims to evaluate the effects of different types of Tai Chi exercises on anxiety and depression in older adults through network meta-analysis, to determine the optimal intervention measures.

## 2 Systematic review

### 2.1 Materials and methods

We have registered the present study and network meta-analysis in the International Prospective Register of Systematic Reviews (PROSPERO) with the identifier CRD42023442270, and strictly adhere to the Preferred Reporting Items for Systematic Reviews and Meta-Analyses Network Meta-Analysis (PRISMA-NMA) statement (25).

### 2.2 Study search and selection

To conduct a comprehensive search, we utilized multiple databases including PubMed, Web of Science, the Cochrane Library, China National Knowledge Infrastructure (CNKI), and the Chinese Scientific Journal Database (VIP). Our search strategy involved a combination of medical subject headings and free-text terms. The subject headings encompassed various keywords such as Tai Chi, tai ji, elderly individuals, anxiety, depression, and randomized controlled trials. We limited our search to articles published in English and Chinese languages, and the search period covered from the inception of the databases until July 26, 2023.

Additionally, we performed a reference search within the included articles to identify additional studies that met our criteria. For more information on our detailed search strategy, please refer to Appendix 1.

Inclusion criteria: These criteria include (a) elderly individuals aged ≥60 years, (b) various types of Tai Chi used as the intervention in the experimental group, including 24-form Simplified Tai Chi (24-TC), Yang-style Tai Chi (YS-TC), Sun-style Tai Chi (SS-TC), and Tai Chi exercise programs (The trials will be classified as TCEP when patients engage in specially designed Tai Chi exercises conducted by the researchers during the Tai Chi exercise) with the control group receiving either a waiting list, usual care, or a different therapy than the aforementioned interventions, (c) the study reports anxiety or depression symptoms as one of the outcome measurement indicators. Validated standardized measurement tools must be used to assess these outcomes. The study must record the results before and immediately after the therapy, and (d) all studies must be randomized controlled trials.

Exclusion criteria: (1) Unclear intervention methods or interventions that combine Tai Chi with other exercises; (2) studies without analyzable data, such as those that do not report values (i.e., mean, standard deviation, and sample size) necessary for calculating effect sizes; (3) participants with unclear age description.

### 2.3 Data extraction and quality assessment

To eliminate duplicate search results, we utilized the EndNote X9 software. Subsequently, the titles and abstracts of the articles were evaluated by two reviewers to ascertain their suitability for inclusion in the study. No further examination was performed for studies that did not meet the established inclusion criteria. The remaining studies that were not excluded underwent a full-text evaluation by the two reviewers (Yangjian Dong and Xiaoqin Kuang). Any discrepancies or uncertainties were resolved through a comprehensive discourse with a third assessor (Jiefu Yang). Two reviewers independently utilized standardized data collection forms to gather comprehensive information, encompassing various aspects. This information included the primary authors’ names, publication year, sample size, gender distribution, age distribution, types of Tai Chi, control group interventions, intervention frequencies, intervention durations, primary outcome measurement indicators, and specific measurement tools employed. Following this, the quality of RCTs was evaluated by two reviewers (Lili Dong and Guodong Chao) using the Cochrane Risk of Bias Assessment (26). The assessment categorized bias risk into three levels: high risk, low risk, or uncertain risk based on the following criteria: (1) Random sequence generation. (2) Allocation concealment. (3) Blinding of participants and personnel. (4) Blinding of outcome assessment. (5) Incomplete outcome data. (6) Selective reporting. (7) Other bias, in situations marked by disagreement, efforts were made to achieve consensus through the process of consultation between the two specialists. Should consensus prove elusive, the involvement of a third expert was sought to render the ultimate decision (25).

### 2.4 Data synthesis and analysis

During the data analysis process, statistical analysis was conducted using Stata 15 software. To address the variation in the anxiety and depression rating scales used in different studies, the standard deviation (SMD) was employed as a measure of effect size to aggregate the results. The comparative relationships among various Tai Chi interventions were explored through network meta-analysis (NMA). For each outcome, SMD means, and 95% confidence intervals were calculated. Heterogeneity was assessed using a statistical test, and if significant, a random-effects model was used; otherwise, a fixed-effects model was utilized. A network diagram was created to visually depict the connections between different Tai Chi interventions. The net-meta command in Stata 15 was utilized in conjunction with a frequentist approach to perform the network meta-analyses. To evaluate the potential inconsistency between direct and indirect evidence, a network-side analysis and a design-treatment interaction model were employed. If the *p*-values of the design-treatment interaction model and the network-side analysis exceeded the predetermined significance level of 5%, a consistency model was applied to assess the effect sizes of multiple treatment comparisons (27, 28).

The determination of the relative effectiveness of various intervention measures was accomplished through the examination of the cumulative probability plot and subsequent calculation of the area under the curve. A greater SUCRA value is indicative of a more favorable treatment effect associated with a specific intervention measure (29). To evaluate the dependability of the study findings and the potential influence of study attributes on patients following the intervention, sensitivity analyses were conducted. (30). To evaluate the dependability of the study findings and the potential influence of study attributes on patients following the intervention, sensitivity analyses were conducted. Comparative adjustment funnel plots were employed to appraise the presence of publication bias. If a balanced distribution of effect sizes is observed among the studies included in the analysis, it indicates negligible publication bias in the network meta-analysis.

## 3 Results

### 3.1 Study inclusion and selection

**Figure 1:**
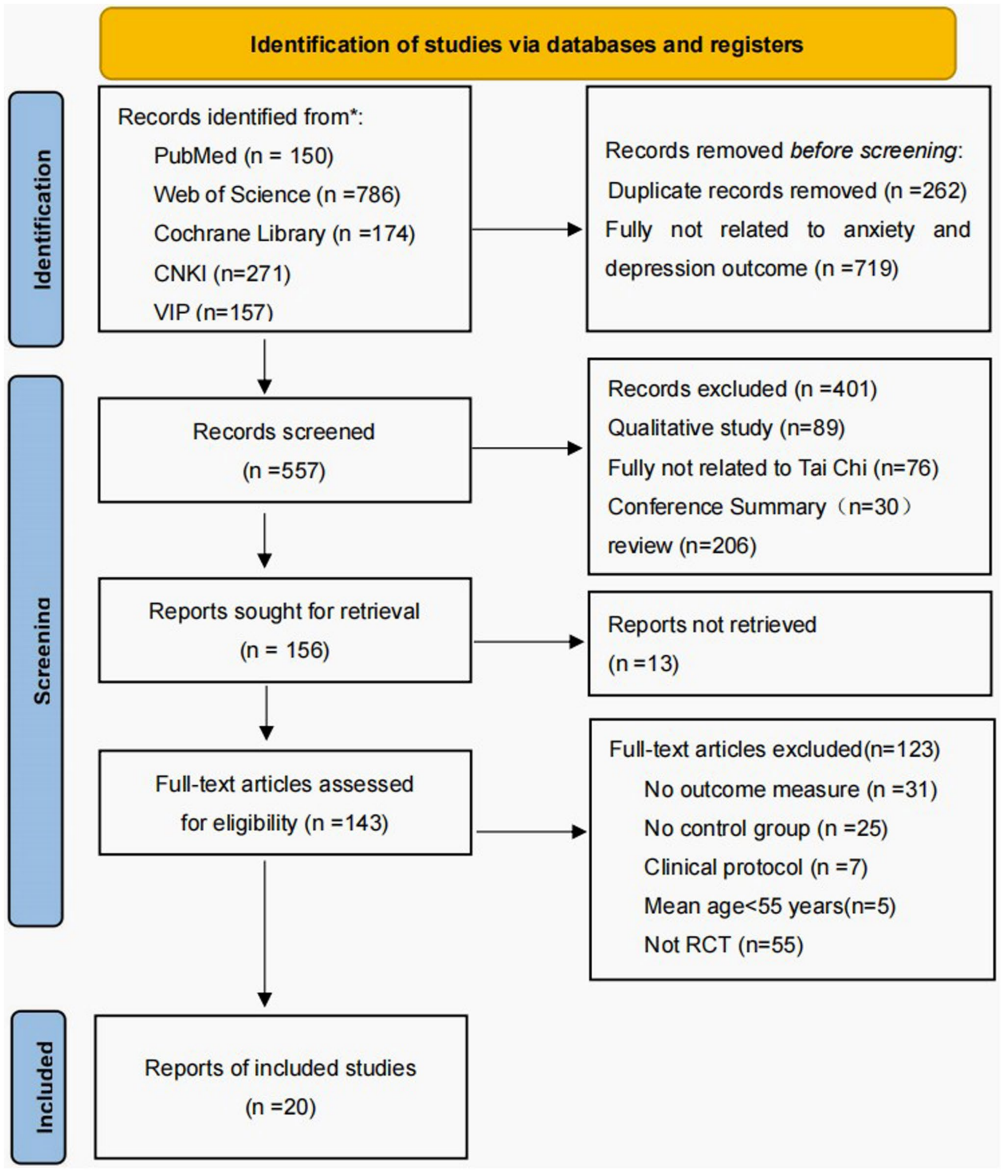
The flow of the study search and selection process.

Using a meticulous and methodical search, we have gathered a total of 20 RCTs from five databases, encompassing 1,798 older participants. Following the elimination of duplicate sources and irrelevant literature about the study subject, we proceeded with an initial evaluation of titles and abstracts, resulting in the inclusion of 557 papers, while 401 papers that did not adhere to the inclusion criteria were excluded. The remaining 156 works were meticulously examined in their entirety, leading to the exclusion of 136 citations that were deemed irrelevant to the study. Moreover, 13 articles were included despite the unavailability of their full texts, while 31 articles were excluded as they did not report the results of the studies. Additionally, 25 articles did not provide information about the control group, 7 articles were part of a clinical guideline, 5 articles involved participants <60 years, and 55 articles were categorized as nonrandomized controlled trial studies. Ultimately, in this network meta-analysis, we have incorporated 20 published randomized controlled trials to compare the efficacy of various Tai Chi interventions against a control group, focusing on the enhancement of anxiety and depressive symptoms in older adults. Figure 1 provides a comprehensive presentation of the selection process for the included studies.

### 3.2 The characteristics of studies

These participants were allocated randomly into experimental and control groups, by a well-established protocol. The experimental group was subjected to four distinct interventions, namely 24-TC, TCEP, YS-TC, and SS-TC. Of these interventions, seven studies focused on 24-TC, eight studies on YS-TC, three studies on the TCEP, and two studies on SS-TC. The duration of each intervention varied between 20 and 90 min, with a frequency ranging from one to five times per week. Conversely, the control group received interventions such as routine daily activities, health education, physical exercise, and usual care. The studies included in this analysis were predominantly conducted in China and were published between 2015 and 2022. Anxiety symptoms were evaluated using the Death anxiety scale, Self-rating Anxiety Scale, Hospital Anxiety and Depression Scale, and Hamilton Rating Scale of Anxiety, while depressive symptoms were assessed using the Geriatric Depression Scale, Self-rating depression Scale, The 21-item Beck Depression Inventory−1A, Cornell Scale for Depression in Dementia, Center for epidemiologic studies depression scale, Hospital Anxiety and Depression Scale, Inventory of Depressive Symptomatology, Hamilton Rating Scale of Depression, Hamilton Rating Scale of Depression, and Cornell Medical Index. Within these 20 randomized controlled trials, Table 1 summarizes the fundamental characteristics of the selected studies.

**Table 1:** Detailed characteristics of each of the included literature studies.

Study	Country	Sample size		Female ratio		Mean age years		Intervention		Frequency and period	Outcome measures	Outcome
		E	C	E	C	E	C	E	C			
Bonab and Parvaneh (2022) (31)	Iran	35	35	100%		60–70		24-TC	routine daily activities	three 60-min sessions/12 weeks	DAS	Anxiety
Liao, et al. (2018) (32)	China	55	52	65%	58%	71.8 ± 7.29	71.75 ± 8.20	YS-TC	health education	three 50 min sessions/3 months	GDS	depression
Ge, et al. (2022) (33)	China	32	33	34.4%	51.5%	70.16 ± 5.40	72.91 ± 6.61	YS-TC	routine daily activities	three 60-min sessions/8 weeks	GDS	depression
Song, et al. (2022) (18)	China	20	20	100%		64.15 ± 8.56	64.15 ± 8.56	TCEP	health education	three 60-min sessions/12 weeks	SAS.SDS	Anxiety depression
Li, et al. (2019) (34)	China	163	163	55.8%	58.3%	63.61 ± 6.62	65.44 ± 5.79	YS-TC	physical exercise	seven 60-min sessions/6 months	SAS.SDS	Anxiety depression
Redwine et al. (2020) (35)	USA	24	23	8%	13%	63 ± 9	67 ± 7	YS-TC	usual care	Two 60-min sessions/16 weeks	BDI-IA	depression
Lam, et al. (2014) (36)	China	171	218	73.1%	78.9%	77.2 ± 6.3	78.3 ± 6.6	24-TC	physical exercise	three 30-min sessions/1 year	CSDD	depression
Hsu, et al. (2016) (37)	China	30	30	63%		80.7 ± 9.68	81.77 ± 6.32	TCEP	routine daily activities	three 40 min sessions /26 weeks	GDS	depression
Huang, et al. (2019) (38)	China	36	38	70%	65%	81.9 ± 6.0	81.9 ± 6.1	TCEP	usual care	three 20 min sessions/10 months	GDS	depression
Chou, et al. (2004) (39)	China	7	7	NA		72.6 ± 4.2		YS-TC	usual care	three 45 min sessions/3 months	CES-D	depression
Ma, et al. (2018) (40)	China	79	79	32%	30%	70.2 ± 10.25	69.7 ± 10.84	24-TC	usual care	Three to five 90 sessions/6 months	CES-D	depression
Leung, et al. (2013) (41)	Australia	19	19	29%		73 ± 8		SS-TC	usual care	Five 30 min sessions/12 weeks	HADS	Anxiety depression
Yeh, et al. (2020) (42)	American	61	31	30%	42%	68.6 ± 9.2	68.1 ± 6.7	YS-TC	health education	Three 30 min sessions/12 weeks	CES-D	depression
Yildirim et al. (2016) (43)	Turkey	30	30	90%	87%	62.9 ± 6.5	64.4 ± 7.5	YS-TC	physical exercise	three1hour sessions/12 weeks	GDS	depression
Solianik et al. (2021) (44)	Lithuania	15	15	87%	77%	≥60		YS-TC	usual care	Two 60 min sessions/10 weeks	HANDS	Anxiety depression
Noradechanunt et al. (2017) (45)	Australia	13	13	69%	87%	66.6 ± 6.7		SS-TC	physical exercise	Two 90 min sessions/24 weeks	CES-D	depression
Yuan et al. (2016) (46)	China	30	30	43%	47%	66.33 ± 5.56	67.47 ± 3.82	24-TC	usual care	Five 20-40 min sessions/12 weeks	HAMD	depression
Liu (2016) (47)	China	32	31	100%		66.3 ± 2.7	65.8 ± 3.2	24-TC	usual care	Five 60 min sessions /16 weeks	SCL-90	Anxiety depression
Liao (2015) (48)	China	40	40	100%		63. 1 ± 4. 20	62. 8 ± 3. 65	24-TC	usual care	Three 60 min sessions /24 weeks	SCL-90	Anxiety depression
Li et al. (2017) (49)	China	30	30	50%		65.83 ± 5.71	56.27 ± 5.40	24-TC	routine daily activities	Five to six 80 min sessions /6 months	HAMA	Anxiety

E, experiment group; C, control group; 24-TC, 24-form Simplified Tai Chi; YS-TC, Yang-style Tai Chi; SS-TC, Sun-style Tai Chi; TCEP, Tai Chi exercise programs; DAS, death anxiety scale; GDS, Geriatric Depression Scale; SAS, Self-rating Anxiety Scale; SDS, Self-rating depression Scale; BDI-IA, The 21-item Beck Depression Inventory − 1A; CSDD, Cornell Scale for Depression in Dementia; CES-D, Center for epidemiologic studies depression scale; HADS, Hospital Anxiety and Depression Scale; IDS-C, Inventory of Depressive Symptomatology; HRSD, Hamilton Rating Scale of Depression; HAMA, Hamilton Rating Scale of Anxiety; HAMD, Hamilton Rating Scale of Depression; CMI, Cornell Medical Index.

**Figure 2:**
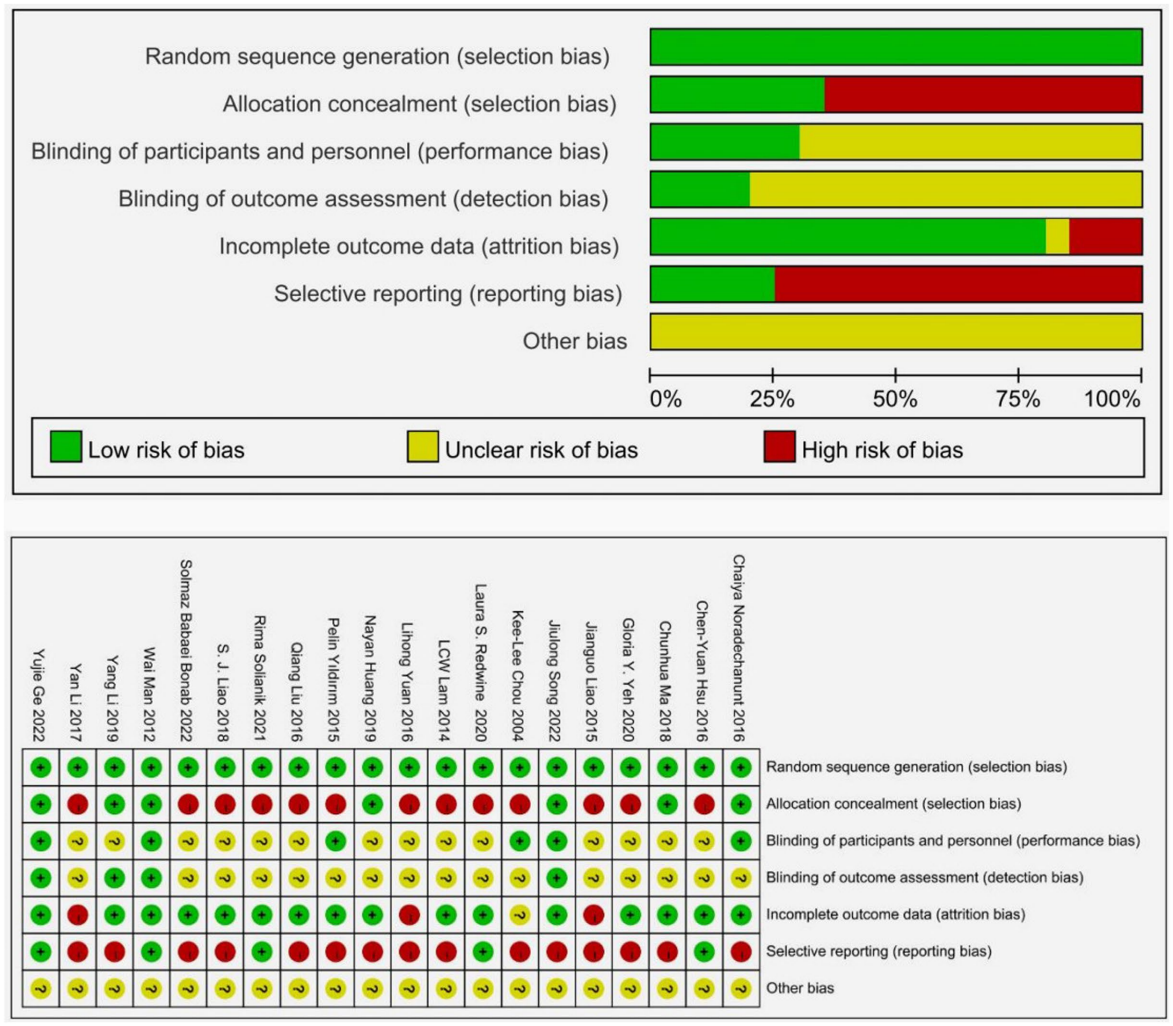
Quality assessment of included studies.

The evaluation of the chosen studies was conducted using the Cochrane Risk of Bias tool, with the findings displayed in Figure 2. Out of the 20 studies included, all were designed with two groups and provided descriptions of the randomization process. Nine studies offered explicit explanations of how the random sequences were generated, while only 7 studies mentioned a specific method of concealing the allocation. Due to the nature of sports interventions, most experiments were not conducted in a blinded manner, with only a few utilizing a single-blind design. The majority of the experiments provided comprehensive reporting on missing data throughout the study, with 16 studies (80%) indicating a low risk of bias regarding incomplete outcome data. None of the studies reported any other forms of bias.

### 3.3 Pairwise meta-analysis

**Figure 3:**
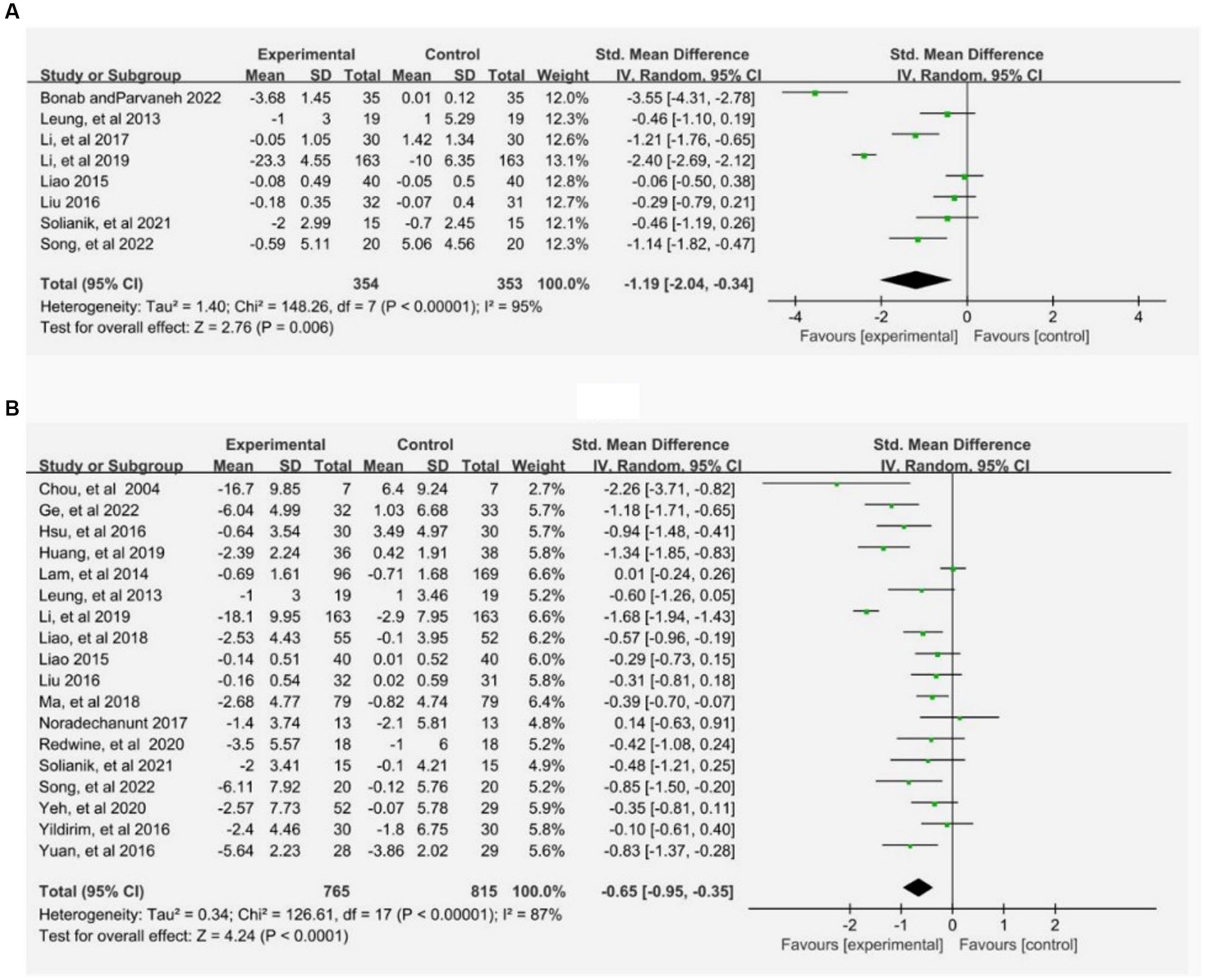
Pairwise meta-analysis of Tai Chi on anxiety **(A)** and depression **(B)**. Meta-analysis results for pair-wise comparisons represented SMD and 95% credible interval.

The disparities between Tai Chi exercise and the control group were examined by conducting a classic paired meta-analysis using a random effects model, after thoroughly analyzing all the interventions in the study. The control group comprised physical exercise, usual care, health education, and routine daily activities. The findings about anxiety demonstrated that Tai Chi intervention exhibited greater than the control group (SMD: −1.19, 95% CI: −2.04, −0.34, *p* < 0.05). Tai Chi was superior to routine daily activities (SMD: −1.73, 95% CI: −3.39, −0.07, *p* < 0.05), health education (SMD: −1.14, 95% CI: −1.82, −0.47, *p* < 0.05), physical exercise (SMD: −2.40, 95% CI: −2.69, −2.12, *p* < 0.05) Similarly, the results concerning depression indicated that Tai Chi exercise was statistically more effective than the control group (SMD: −0.65, 95% CI: −0.95, −0.65, *p* < 0.05). Tai Chi was superior to routine daily activities (SMD: −1.18, 95% CI: −1.71, −0.65, *p* < 0.05), health education (SMD: −0.54, 95% CI: −0.81, −0.27, *p* < 0.05), usual care (SMD: −0.66, 95% CI: −0.93, −0.40, *p* < 0.05) The comprehensive results of the paired meta-analysis can be observed in the charts (A) and (B) below, and the respective comparison results can also be found in the Supplementary materials (Figure 3).

### 3.4 Network meta-analysis

**Figure 4:**
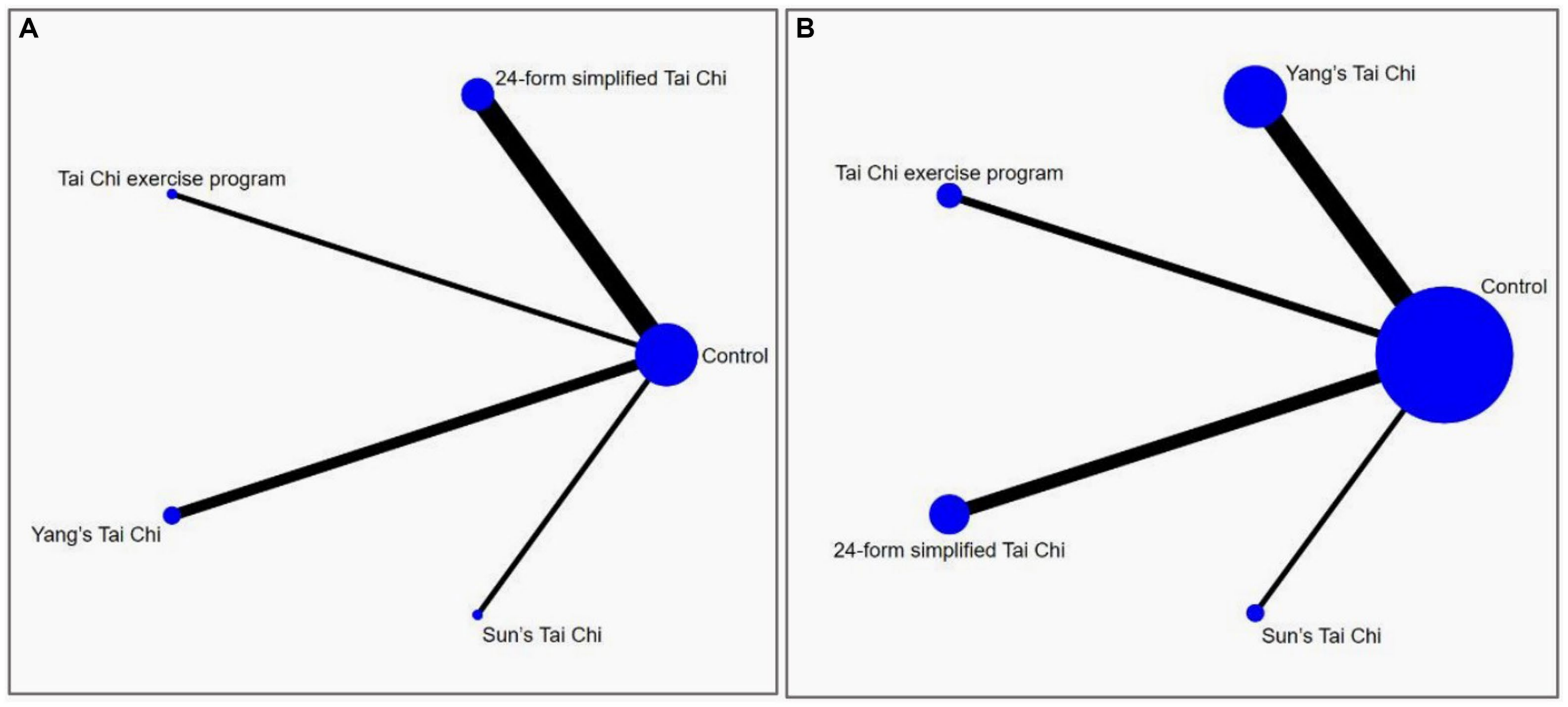
The network structure of the analyzed treatment comparisons for the outcome of anxiety **(A)** and depression **(B)**.

A comprehensive mesh diagram was constructed to examine the relationship between various forms of Tai Chi and their impact on anxiety and depressive symptoms in older adults, based on the analysis of 20 relevant studies (Figure 4). The graph visually represents the connections between the different studies, with lines indicating direct comparisons and dots representing randomized controlled trials that have not yet been directly compared. The thickness of the connecting lines reflects the number of studies that have conducted comparisons between the respective interventions. To ensure the reliability of the findings, consistency analyses were conducted to assess the anxiety and depression outcome measures used in the included studies. The results indicated that all PSRF parameter values exceeded 0.05 and were close to 1.00, suggesting a high level of convergence in the model. Consequently, a reticulated Meta-analysis was conducted given the presence of a consistent model.

**Figure 5:**
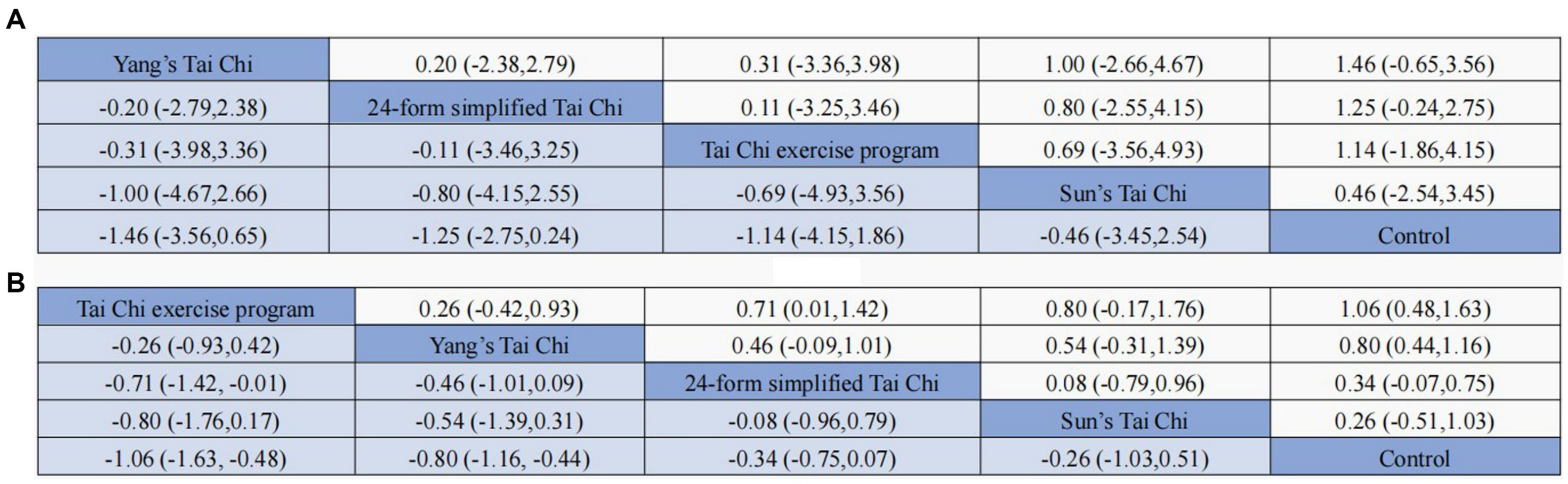
Results of the network meta-analysis (SMD vs. 95% CI) of the effects different TCM exercise on anxiety **(A)** and depression **(B)** in the adults.

**Figure 6:**
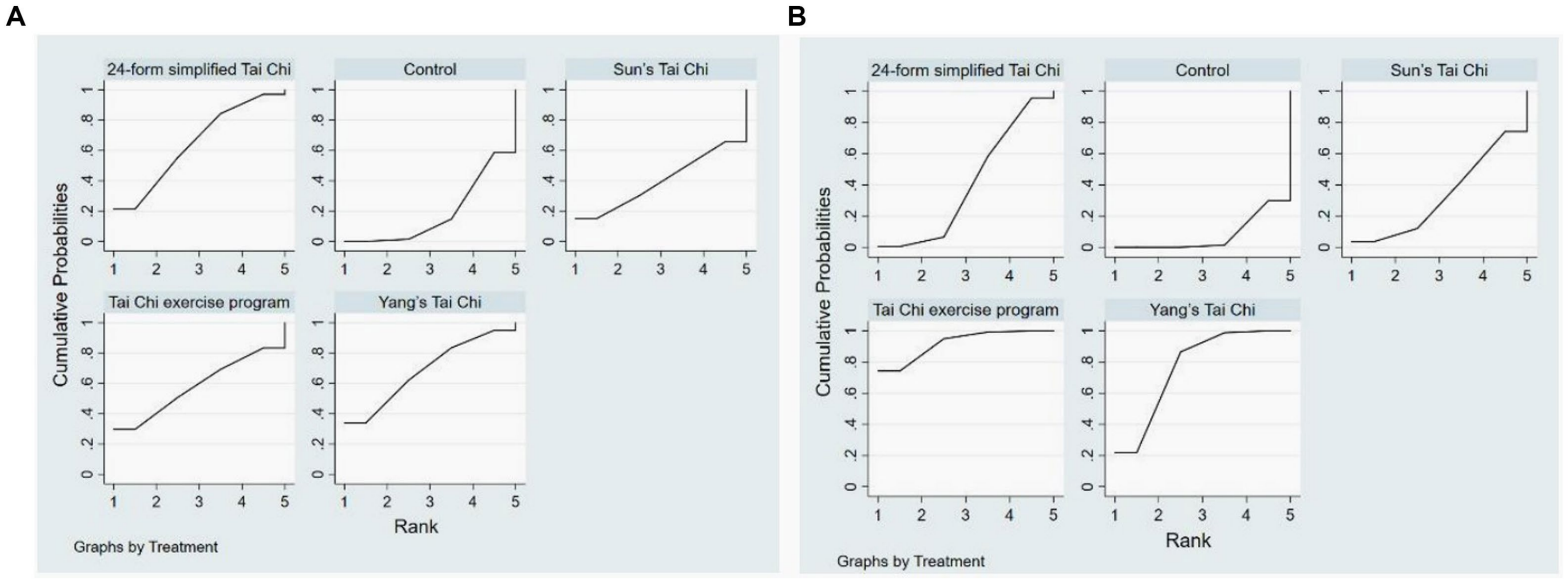
Anxiety **(A)** and **(B)** depression rank probability.

The network meta-analysis encompassed eight studies that examined four distinct forms of Tai Chi interventions. These interventions were compared to a control group in terms of their impact on anxiety symptoms, with a total of 707 subjects participating in the analysis. As simultaneous direct and indirect comparisons were not present in the network diagram, a consistency model was employed to assess the different interventions. Figure 5A displays the specific findings in comparison to the control group. YS-TC (SMD: −1.46, 95% CI: −3.56, 0.65), 24-TC (SMD: −1.25, 95% CI:2.75, 0.24), TCEP (SMD: −1.14, 95% CI: −4.15, 1.86), and SS-TC (SMD: −0.46, 95% CI: −3.45, 2.54) all demonstrated superior outcomes. Additionally, Figure 6A presents a probability ranking table indicating that YS-TC exercise possessed the highest likelihood (69.9%) of being considered the most effective intervention for anxiety in older adults, closely followed by 24-TC (66.8%).

In Figure 4B, the network diagram depicting depressive symptoms among 1,580 subjects is presented, encompassing 18 studies and 4 distinct interventions. Given the absence of concurrent direct and indirect comparisons within the network diagram, a consistency model was employed to evaluate the various interventions. The outcomes illustrated in Figure 5B indicate that when compared to control groups, TCEP (SMD: −1.06, 95% CI: −1.63, −0.48), YS-TC (SMD: −0.80, 95% CI: −1.16, −0.44), 24-TC (SMD: −0.34, 95% CI: −0.75, –0.07), and SS-TC (SMD: −0.26, 95% CI: −1.03, 0.51) all exhibited superior efficacy. Furthermore, the probability ranking table in Figure 6B reveals that TCEP is deemed the most probable optimal intervention for depression in older adults (92.6%), followed by YS-TC (77.9%).

### 3.5 Publication bias

**Figure 7:**
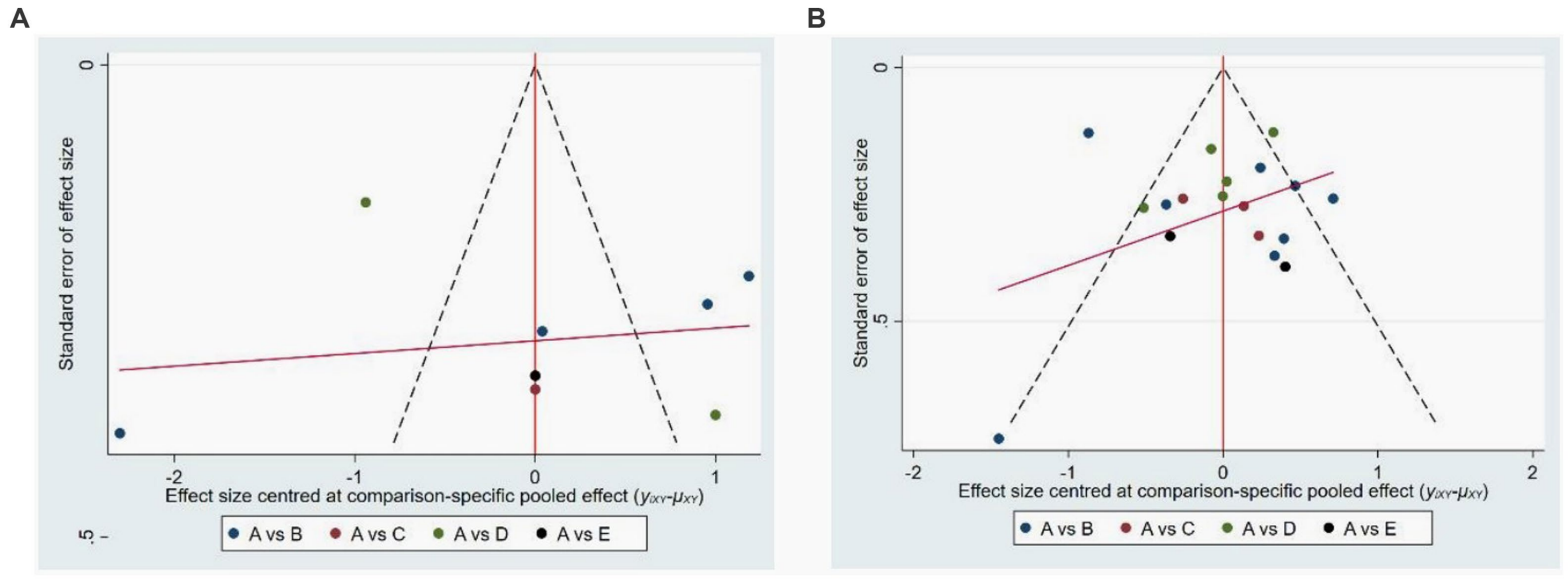
Publications bias of four tyles of Tai Chi on anxiety **(A)** and depression **(B)**. In the picture **(A)**, A is control group, B is 24-form simplified Tai Chi, C is Tai Chi exercise program, D, is Yang style Tai Chi, E is Sun style Tai Chi. In picture **(B)**, A is control group, B is Yang style Tai Chi, C is Tai Chi exercise program, D is 24-form simplified Tai Chi, E is Sun style Tai Chi.

Among the 20 studies ultimately included in the analysis, seven of them extensively examined the effects of various forms of Tai Chi practice on anxiety symptoms in elderly individuals, while another 18 studies reported on the effects of these exercises on depressive symptoms. All of these studies were assessed to have a moderate to high level of quality. Additionally, we utilized STATA software version 15 to create funnel plots, using anxiety and depression as indicators of study outcomes. The results revealed that the funnel plot for depressive symptoms (Figure 7B) displayed a predominantly stacked shape, suggesting the absence of significant publication bias for the depression indicator. However, the funnel plot for anxiety symptoms (Figure 7A) exhibited some degree of asymmetry, which publication bias may be due to the small number of studies included and the insufficient sample size of some Tai Chi.

### 3.6 Sensitivity analysis

Through the implementation of sensitivity analysis, it was determined that the exclusion of individual studies did not result in any substantial alteration to the statistical significance of the primary or secondary outcomes. This additional analysis serves to confirm the strength and reliability of the findings, as well as the diversity within the study.

## 4 Discussion

The efficacy of Tai Chi in alleviating anxiety and depressive symptoms among older adults has been well-documented. (20). Our research endeavors to thoroughly examine the existing body of literature on Tai Chi interventions for anxiety and depression. While previous studies have investigated this area, they have not definitively determined the most effective form of Tai Chi for addressing these mental health concerns. Consequently, our study employed a network meta-analysis methodology to evaluate the impact of various types of Tai Chi interventions on anxiety and depression symptoms in older adults. All of Our comprehensive analysis encompassed different variants of Tai Chi against a control group. Based on our meticulous analysis of the research data, we have ranked the effects on anxiety from most significant to least as follows: YS-TC, 24-TC, TCEP, SSTC, CC. Similarly, for depression, the intervention effects were ranked as follows: TCEP, YS-TC, 24-TC, SS-TC, and CC. These findings underscore the potential of specific forms of Tai Chi, such as YS-TC and TCEP, in significantly ameliorating anxiety and depression among elderly individuals.

Compared with the control group that did not exercise or routine daily activities, Tai Chi exercise, with its characteristics of low intensity and safety, can improve anxiety and depression symptoms and enhance the quality of life in older adults. This conclusion is consistent with previous systematic review studies, which consider Tai Chi exercise as one of the effective methods for treating anxiety and depression (20, 50). This effect may be related to the reduction of sympathetic nervous system activity caused by Tai Chi exercise. Researchers have found that Tai Chi exercise can produce certain specific cell mediators, such as transforming growth factor-β and interleukin-10, by measuring cortisol levels in saliva. The production of these mediators helps improve the quality of life and reduce psychological stress (51). In addition, Tai Chi exercise can also induce changes in the brain, which may enhance participants’ ability to cope with anxiety and depression, thereby improving patients’ emotional states (52). However, studies have shown that compared with physical exercise, the effect of Tai Chi exercise on improving depression in older adults is not significant. This may be because physical exercise is simpler and easier to obtain basic skills compared to Tai Chi exercise. At the same time, due to the complexity of Tai Chi exercise, participants are more likely to give up in unsupervised situations (45).

Regarding indicators of anxiety, YS-TC exhibits the most effective intervention effect, followed by 24-TC and TCEP. YS-TC offers a smooth rhythm and gentle movements, rendering it suitable for individuals of diverse ages and physical conditions to partake in moderate exercise (53). The participants in this study were elderly individuals presenting anxiety symptoms, hence YS-TC displayed the most favorable effect in terms of anxiety indicators. 24-TC is a widely practiced routine that was simplified and adopted by the Chinese National Sports Commission in 1956, based on the traditional Yang-style Tai Chi (34, 54). The techniques have been simplified, resulting in alterations in the intensity and difficulty of the practice, making it more accessible to the general public. However, in terms of addressing anxiety symptoms, the effect of 24-TC is slightly less than that of Yang-style Tai Chi. According to the ranking results, there is minimal difference in the probability ranking of the effects of YS-TC and 24-TC, with percentages of 69.9 and 66.8%, respectively. Therefore, to determine the optimal treatment plan, it is imperative to consider the severity of anxiety and other factors, facilitating the provision of a more precise individualized treatment plan.

Regarding indicators of depression, TCEP exhibits the most effective intervention effects, with Yang-style Tai Chi following closely behind. Due to the intricate nature of depression’s origins, a generic form of Tai Chi may not be universally applicable. Consequently, researchers have devised specialized TCEPs that cater to individual needs by modifying and adapting movements, thereby better addressing personalized requirements, reducing physical ailments, and ameliorating depressive symptoms (18, 37, 38). In contrast, Yang-style Tai Chi, characterized by its diverse range of movements, can be selected and practiced by various populations, effectively enhancing physical functioning and promoting social engagement to alleviate depressive symptoms (55). Furthermore, Yang-style Tai Chi remains prominently positioned in the treatment of depressive symptoms among the elderly population, as evidenced by its significant probability ranking.

The research findings indicate that SS-TC has significant effects in mitigating depressive symptoms (36), which aligns with our research outcomes. Nonetheless, these two variants of Tai Chi Chuan received lower rankings in terms of anxiety and depression outcome measures. This discrepancy can be attributed to the fact that Sun-style Tai Chi Chuan falls under the category of small frame postures, characterized by smaller and more compact movements that excel in martial arts performance. Such postures necessitate agility, fluidity in movement, and the coordination of hands, eyes, body, techniques, and steps (45, 56). Consequently, these attributes render this particular form of Tai Chi less suitable for the elderly population, especially those who are ailing or have limited mobility.

Overall, our research has certain clinical implications. Firstly, our research provides evidence to support the benefits of tai chi in alleviating anxiety and depression symptoms in older adults. Through a systematic review and analysis of different tai chi interventions, our study offers valuable information for tai chi as a treatment option for anxiety and depression in older adults. Secondly, our study addresses the lack of research comparing the clinical effectiveness of different tai chi techniques in managing anxiety and depression symptoms in older adults. By comparing four different tai chi intervention methods, our study contributes to understanding the relative efficacy of different forms of tai chi in improving psychological health outcomes. Lastly, our study utilizes traditional meta-analysis and network meta-analysis methods to analyze the data. The combination of these two methods allows for a more comprehensive evaluation of the included studies and quantitatively assesses the effectiveness of tai chi interventions. Therefore, these evidence-based findings can be recommended to older adults experiencing anxiety and depression symptoms, providing more accurate options for non-pharmacological treatments to clinical practitioners.

Undoubtedly, our study possesses certain limitations. Firstly, in the traditional Meta-analysis phase, we observed substantial heterogeneity, which may be attributed to variances in intervention duration, frequency, and duration among different intervention groups in the included studies. Additionally, the utilization of diverse intervention measures in the control groups could also serve as a potential source of heterogeneity. Fortunately, in the network Meta-analysis, we did not observe any closed loops, indicating the absence of inconsistency or publication bias in the network Meta-analysis. Secondly, due to Tai Chi being a non-pharmacological therapeutic physical exercise, it was not feasible to implement complete blinding of participants during the intervention, thereby potentially introducing bias into the study results. Third, there exist numerous styles and forms of Tai Chi, yet our study exclusively incorporated a subset of Tai Chi styles to examine their effects on anxiety and depression symptoms in older adults. Research with fewer databases, a limited number of included articles, and relatively small sample sizes may introduce bias to the research findings. Fourthly, this study did not adequately consider sleep as one of the factors affecting exercise effectiveness and depressive symptoms (57, 58). In future research exploring the impact of physical activity or exercise on depressive symptoms, the influence of sleep should be fully considered. Fifthly, this network meta-analysis only focused on older participants, therefore caution should be exercised when generalizing these conclusions to other populations.

In subsequent investigations, it is imperative to concentrate on augmenting the impartiality, uniformity, and overall excellence of the experimental structure. When choosing literature for analysis, preference should be afforded to studies with extensive participant samples and forward-looking research designs. Additionally, it is significant to take into account literature that encompasses diverse variations of Tai Chi, including Ai Chi, Chen-style Tai Chi, and Wu-style Tai Chi, as well as literature encompassing an array of Tai Chi forms and styles. This methodology aids in broadening the range of intervention measures, reducing research bias, and facilitating the precise selection of the most efficacious Tai Chi interventions.

## 5 Conclusion

The findings from the network meta-analysis conducted in this study demonstrate that the practice of Tai Chi exerts a noteworthy and favorable influence on the enhancement of anxiety and depressive symptoms among the elderly population. Among the various studies analyzed, it was found that Yang-style Tai Chi specifically demonstrated notable advancements in alleviating symptoms of anxiety. For older individuals who are seeking ways to ameliorate symptoms of depression, we suggest considering Tai Chi exercise programs as a viable treatment option. However, healthcare professionals need to consider individual variations among patients to scientifically select an appropriate exercise regimen. Consequently, this research provides clinical practitioners with valuable evidence and guidance regarding non-pharmacological treatment options that can enhance the well-being of elderly individuals coping with anxiety and depression.

## Data availability statement

The primary contributions of this research are outlined in the article/Supplementary materials, and for additional inquiries, you can reach out directly to the corresponding author.

## Author contributions

XK: Data curation, Methodology, Software, Validation, Writing – original draft. YD: Data curation, Methodology, Software, Validation, Writing – original draft. LS: Project administration, Supervision, Writing – review & editing. LD: Data curation, Software, Writing – original draft. GC: Data curation, Software, Writing – original draft. XZ: Writing - review & editing, Methodology, Supervision, Formal analysis. JY: Formal analysis, Methodology, Project administration, Writing – review & editing.
