# Supplementary material for: The effects of different types of Tai Chi exercise on anxiety and depression in older adults: a systematic review and network meta-analysis
Source: Front Public Health. 2024 Jan 8;11:1295342. doi: 10.3389/fpubh.2023.1295342 (PMC10800705; doi:10.3389/fpubh.2023.1295342)
Supplement: Supplementary file 1 [file Data_Sheet_1.docx]

Supplementary Material

Search strategy

PubMed

| **Step** | **Search strategy** |
| --- | --- |
| #1 | (Aged[MeSH Terms]) OR (elderly[Title/Abstract]) |
| #2 | ((((((((((Tai Ji[MeSH Terms]) OR (Tai-ji[Title/Abstract])) OR (Tai Chi[Title/Abstract])) OR (Chi, Tai[Title/Abstract])) OR (Tai Ji Quan[Title/Abstract])) OR (Ji Quan, Tai[Title/Abstract])) OR (Quan, Tai Ji[Title/Abstract])) OR (Taiji[Title/Abstract])) OR (Taijiquan[Title/Abstract])) OR (T'ai Chi[Title/Abstract])) OR (Tai Chi Chuan[Title/Abstract]) |
| #3 | (((randomized controlled trial[Publication Type]) OR (controlled clinical trial[Publication Type])) OR (randomized[Title/Abstract])) |
| #4 | #1 AND #2 AND #3 |

Cochrane

| Step | Search strategy |
| --- | --- |
| #1 | MeSH descriptor: [Aged] explode all trees |
| #2 | (elderly):ti,ab,kw |
| #3 | #1 OR #2 |
| #4 | MeSH descriptor: [Tai Ji] explode all trees |
| #5 | (Chi, Tai):ti,ab,kw OR (Taijiquan):ti,ab,kw OR (Taiji):ti,ab,kw OR (T'ai Chi):ti,ab,kw OR (Tai Chi):ti,ab,kw OR (Ji Quan, Tai):ti,ab,kw OR (Tai Chi Chuan):ti,ab,kw OR (Tai Ji Quan):ti,ab,kw OR (Quan, Tai Ji):ti,ab,kw OR (Tai-ji):ti,ab,kw |
| #6 | #4 OR #5 |
| #7 | (randomized controlled trial):pt OR (controlled clinical trial):pt OR (randomized):ti,ab,kw |
| #8 | #3 AND #6 AND #7 |

Web of Science

| Step | Search strategy |
| --- | --- |
| #1 | (TS=(Aged)) OR TS=(elderly) |
| #2 | ((((((((((TS=(Tai Ji)) OR TS=(Tai-ji)) OR TS=(Tai Chi)) OR TS=(Chi, Tai)) OR TS=(Tai Ji Quan)) OR TS=(Ji Quan, Tai)) OR TS=(Quan, Tai Ji)) OR TS=(Taiji)) OR TS=(Taijiquan)) OR TS=(T'ai Chi)) OR TS=(Tai Chi Chuan) |
| #3 | ((TS=(randomized controlled trial)) OR TS=(controlled clinical trial)) OR TS=(randomized) |
| #4 | #1 AND #2 AND#3 |

China National Knowledge Infrastructure (CNKI)

| Step | Search strategy |
| --- | --- |
| #1 | (SU % '太极' OR SU % '太极拳') AND (SU % '老年人' OR SU % '老人' OR SU % '年老者') |

The Chinese Scientific Journal Database (VIP)

| Step | Search strategy |
| --- | --- |
| #1 | (M=太极 OR M=太极拳) AND (M=老年人 OR M=老人 OR M=年老者) |

**Table 2 Risk of Bias Assessment**

| Study | Random sequence | allocation hiding | Study object blind method | Results evaluation blind method | Loss of follow-up description | Reporting bias | Other bias |
| --- | --- | --- | --- | --- | --- | --- | --- |
| Bonab and Parvaneh 2022 | low | high | unclear | unclear | low | high | unclear |
| Liao, et al. 2018 | low | high | unclear | unclear | low | high | unclear |
| Ge, et al. 2022 | low | low | low | low | low | low | unclear |
| Song, et al. 2022 | low | low | low | low | low | high | unclear |
| Redwine, et al. 2020 | low | high | unclear | unclear | low | low | unclear |
| Hsu, et al. 2016 | low | high | unclear | unclear | low | low | unclear |
| Huang, et al.2019 | low | low | unclear | unclear | low | high | unclear |
| Chou, et al. 2004 | low | high | low | unclear | low | high | unclear |
| Study | Random sequence | allocation hiding | Study object blind method | Results evaluation blind method | Loss of follow-up description | Reporting bias | Other bias |
| Ma, et al. 2018 | low | low | unclear | unclear | low | high | unclear |
| Leung, et al.2013 | low | low | low | low | low | low | unclear |
| Yeh, et al.  2020 | low | high | unclear | unclear | low | high | unclear |
| Solianik, et al. 2021 | low | high | unclear | unclear | low | low | unclear |
| Yuan et al.  2016 | low | high | unclear | unclear | high | high | unclear |
| Liu 2016 | low | high | unclear | unclear | low | high | unclear |
| Liao 2015 | low | high | unclear | unclear | high | high | unclear |
| Li, et al. 2019 | low | low | unclear | low | low | high | unclear |
| Lam, et al. 2014 | low | high | unclear | unclear | low | high | unclear |
| Yildirim, et al. 2016 | low | high | low | unclear | low | high | unclear |
| Noradechanunt, et al. 2017 | low | low | low | unclear | low | high | unclear |
| Li et al. 2017 | low | high | unclear | unclear | high | high | unclear |


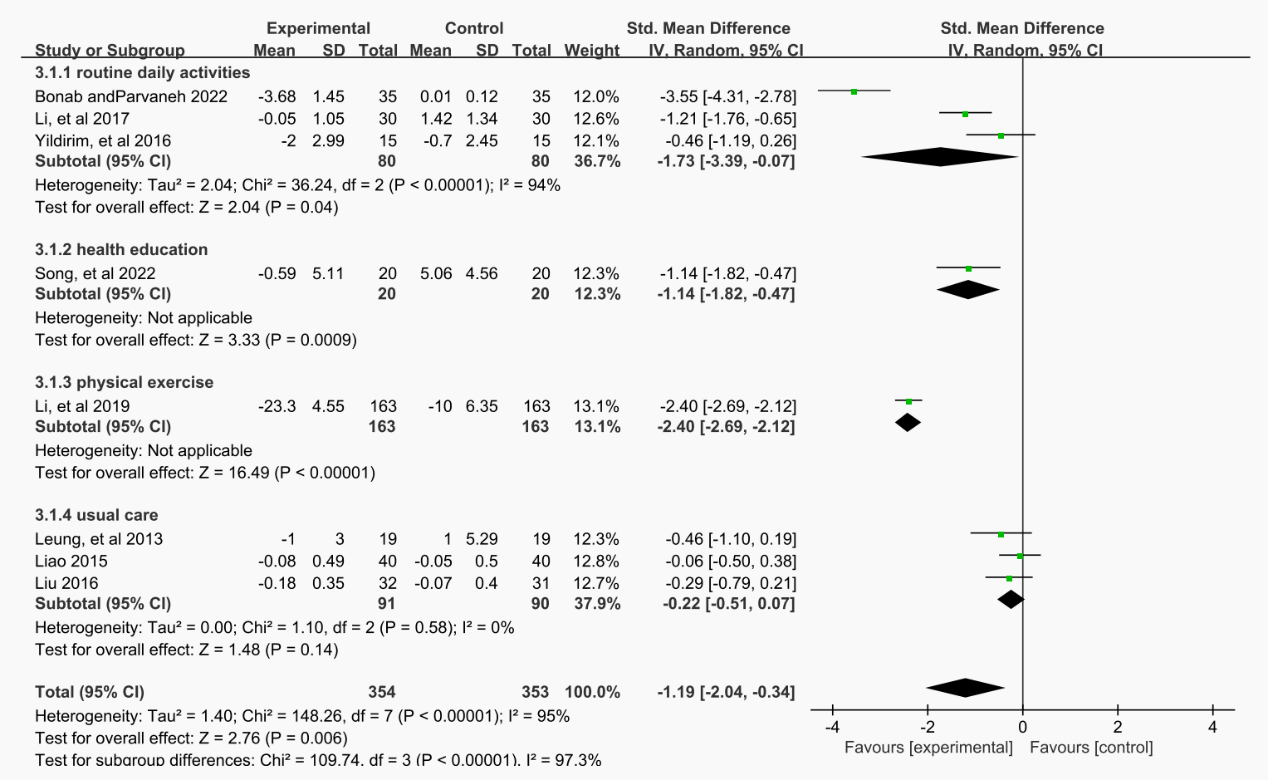


(A)


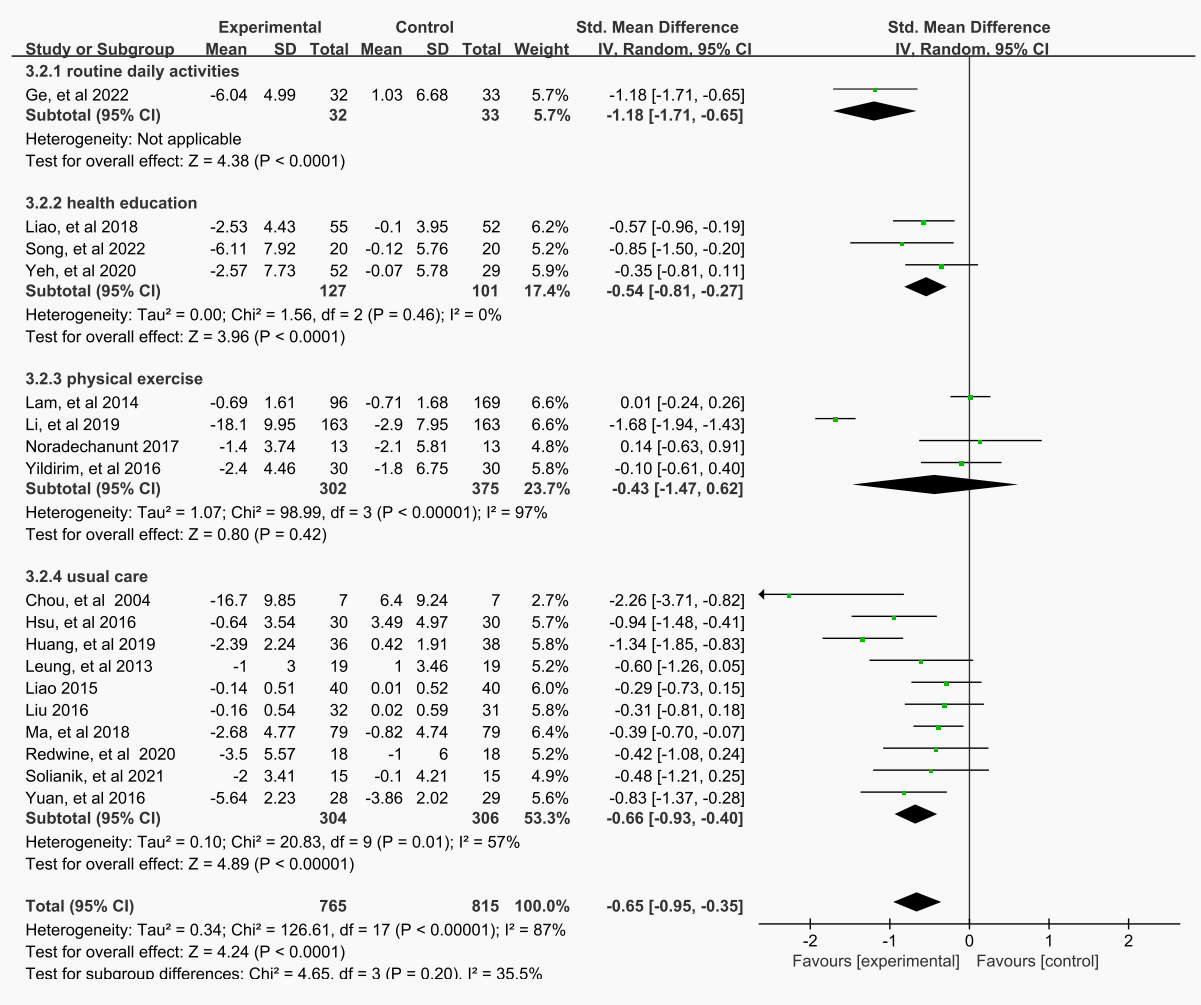


(B)

Figure 8 Pairwise meta-analysis of Tai Chi VS different control groups for anxiety (A) and depression (B).


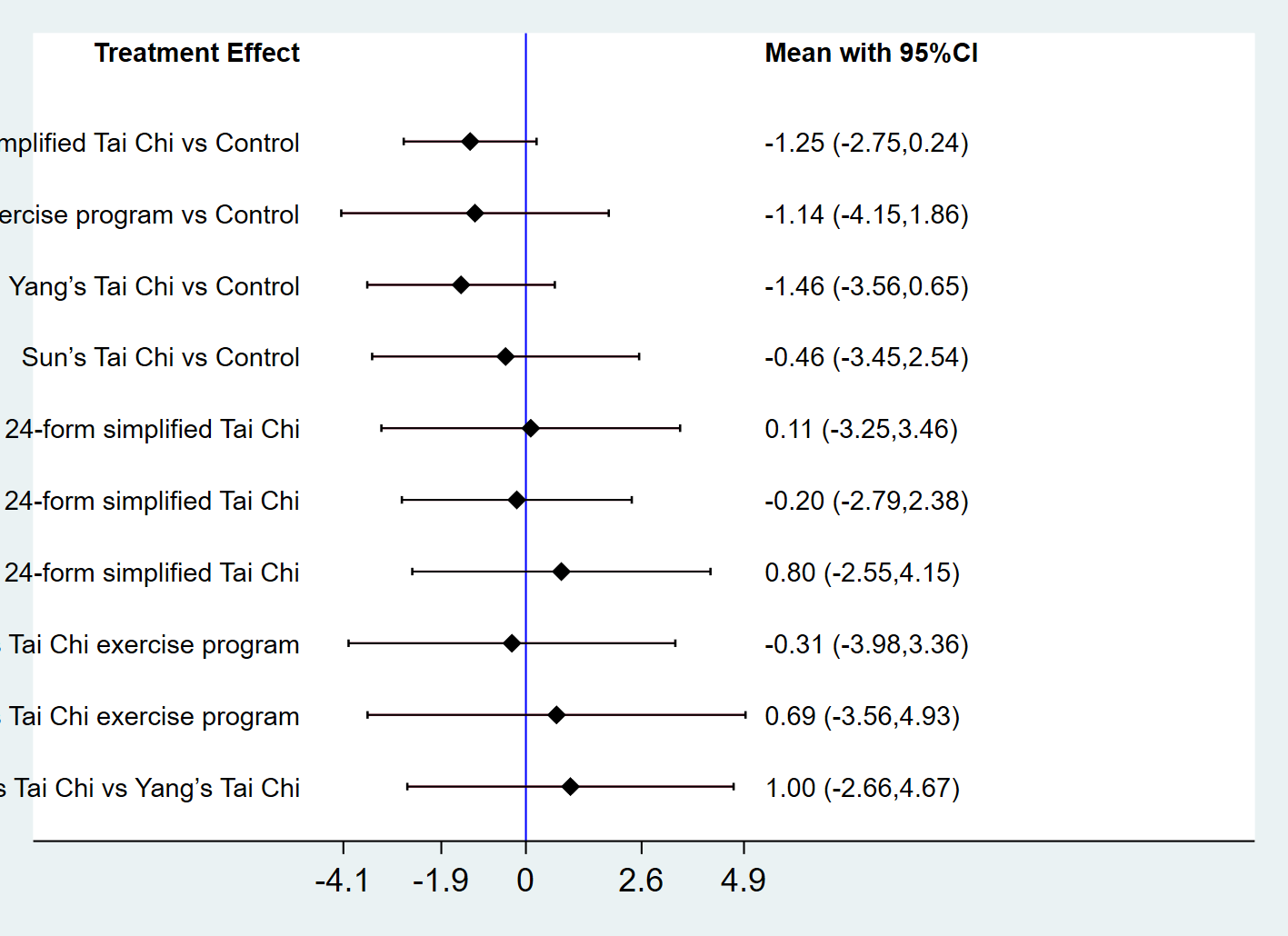


Figure 9 Interval plot of network meta-analysis for anxiety in the elder


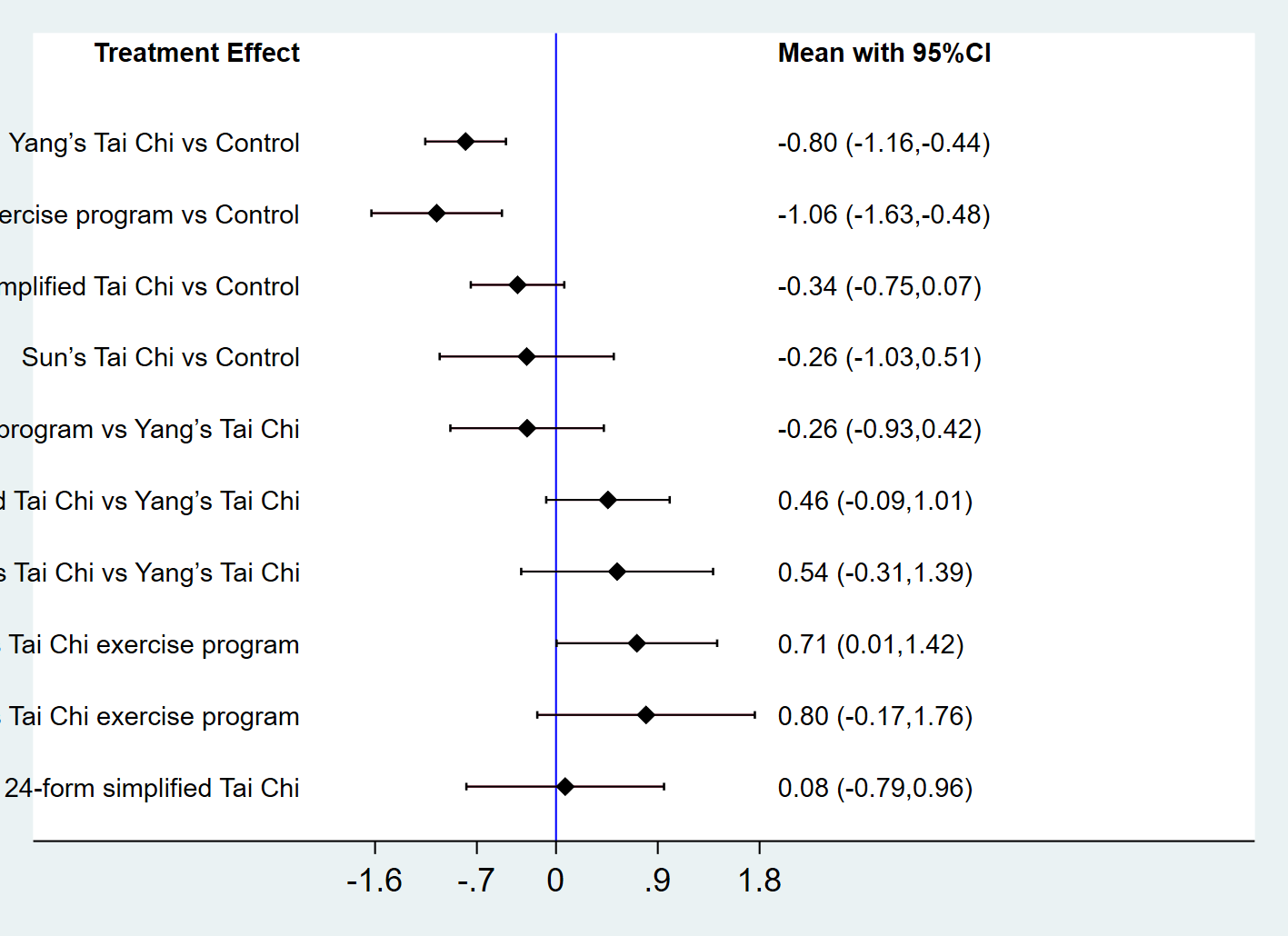


Figure 10 Interval plot of network meta-analysis for depression in the elder


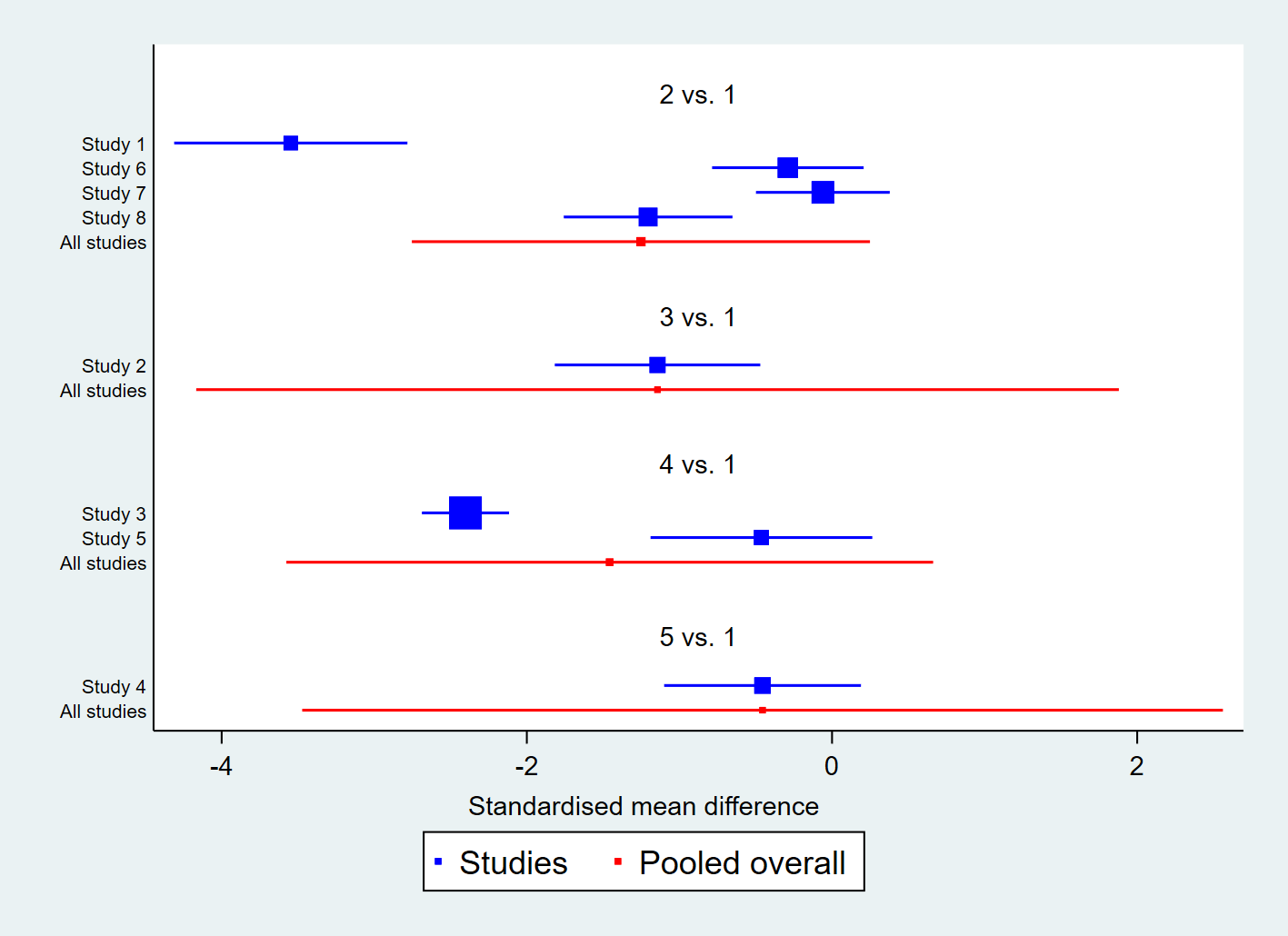


Figure 11 Forest plot of the effect of Tai Chi on anxiety in the elderly


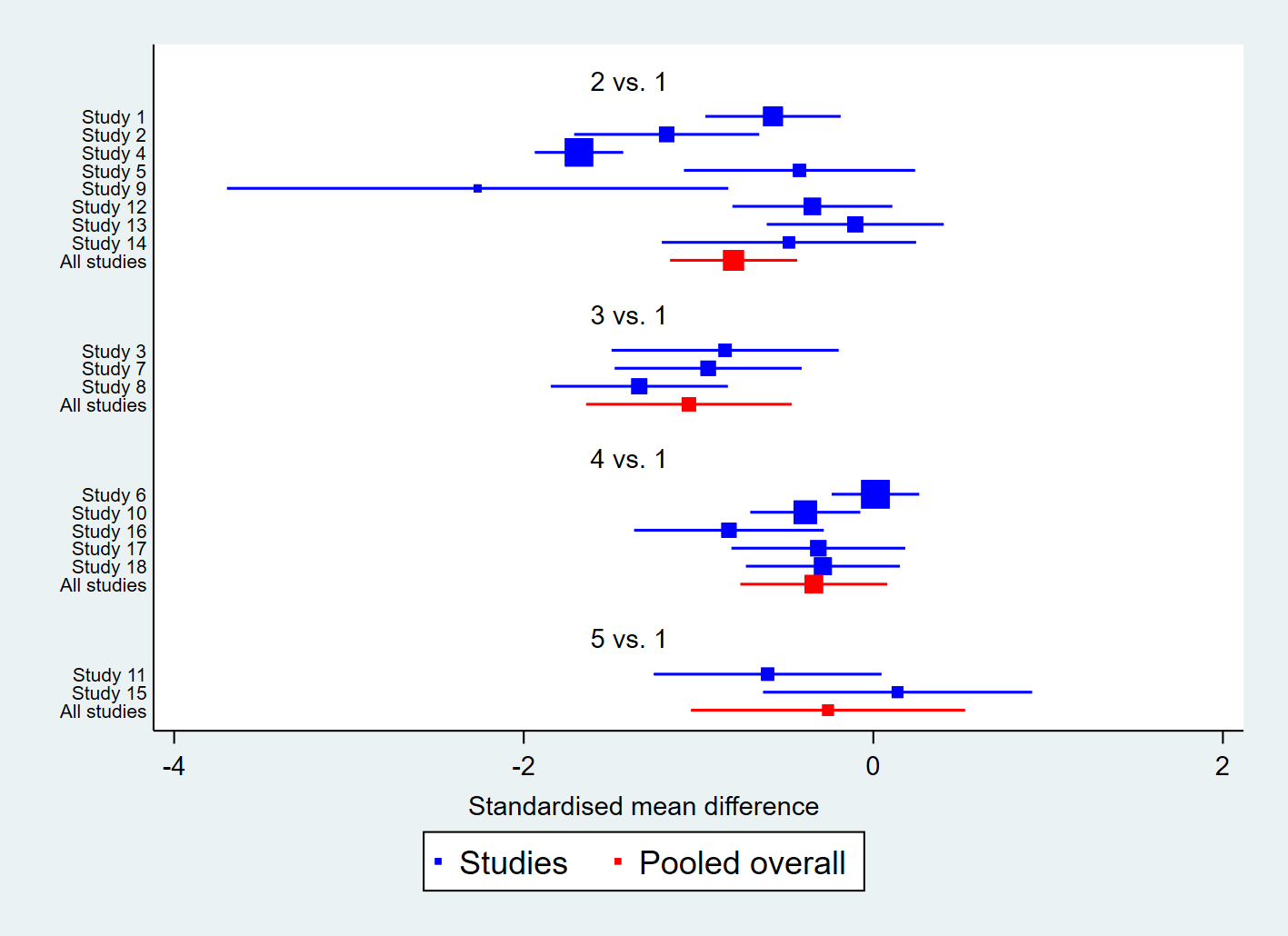


Figure 12 Forest plot of the effect of Tai Chi on depression in the elderly
